# Food Access in New York City During the COVID-19 Pandemic: Social Media Monitoring Study

**DOI:** 10.2196/49520

**Published:** 2025-05-09

**Authors:** Leah Butz, Charles Platkin, Jonathan Chin, Juan Pablo Chavez Salas, Ellie Serres, May May Leung

**Affiliations:** 1 Hunter College NYC Food Policy Center Hunter College New York, NY United States; 2 Hunter College New York, NY United States

**Keywords:** Twitter, food access, food insecurity, New York City, topic modeling, social media, artificial intelligence

## Abstract

**Background:**

The COVID-19 pandemic exacerbated issues of poverty and food insecurity in New York City, and many residents experienced difficulty accessing available resources to help them get food on the table. Social media presents an opportunity to observe and understand the barriers people face in accessing affordable, nutritious, and culturally appropriate foods.

**Objective:**

This study aims to explore the food access discourse during the COVID-19 pandemic on Twitter (subsequently rebranded as X) in New York City by analyzing publicly available tweets posted from March 1, 2020, to March 31, 2021.

**Methods:**

Tweets posted by individuals in New York City during the first 13 months of the COVID-19 pandemic were collected using the observation platform Talkwalker. We categorized a list of multiple keywords into related groups (search strings). Data were cleaned to keep only tweets relevant to food insecurity and food access in New York City and remove duplicate tweets. The software Botometer was used to remove accounts considered to be bots. Topic modeling was used to group these tweets into relevant themes, which were analyzed. The top viral tweets (ie, tweets that received the highest number of retweets in New York City) from this period were further analyzed.

**Results:**

We identified 6 major themes (with subthemes) that emerged from the analysis (in order of popularity): community efforts, public assistance programs, grocery shopping and food workers, school foods, millions go hungry, and food justice. Interesting terms that emerged from the data were also identified. Overall, quantities of tweets increased in correlation with current events, such as the closure of New York City public schools; the expansion of the Supplemental Nutrition Assistance Program and unemployment benefits; the proliferation of mutual aid groups in the spring of 2020; and the May Day Instacart, Amazon, and Target strike in 2020.

**Conclusions:**

Findings revealed that in the earliest months of the COVID-19 pandemic, Twitter users in New York City quickly responded to the wave of need by sharing information and resources about food access in their communities. Some users turned to Twitter to either solicit or offer help finding food. Furthermore, the platform lent itself to many conversations about the policies enacted on a federal, state, and city level to help feed New Yorkers in need. Future research on this topic should include an analysis of social media posting on platforms such as Facebook, as well as languages other than English. Results from this type of research can provide information to community leaders and elected officials to better address future crises.

## Introduction

### Background

The COVID-19 pandemic was one of the causes of a global food emergency that only intensified an already fragile food environment for many of our communities’ most susceptible members [1]. Food banks and soup kitchens saw unprecedented demand, and enrollment in federal assistance programs such as the Supplemental Nutrition Assistance Program (SNAP) and the Supplemental Nutrition Program for Women, Infants, and Children increased [1]. Food supply also decreased dramatically, due in part to new difficulties in transportation and storage [2], all the while emergency food relief providers were faced with increasing operational costs [3]. These struggles disproportionately impacted low-income communities, who were more likely to lose income and reduce spending on food [1,4]. Malnutrition (including both undernutrition and overnutrition) can increase an individual’s risk of chronic diet-related diseases, which in turn can increase the risk of COVID-19 and corresponding adverse health outcomes [5].

New York City specifically saw a major food crisis during the COVID-19 pandemic [6]. Food insecurity and lack of access to affordable, healthy foods persist and exert a disproportionate burden on low-income neighborhoods and racial and ethnic minority communities in New York City. The onset of the COVID-19 pandemic only exacerbated these issues as New York City officials reported that “2 million or more” residents faced food insecurity in May 2020 [7]. This was especially acute among Black people, Indigenous people, and other racial and ethnic minorities [8]. While New York City worked quickly to feed hungry residents, existing infrastructure was not equipped to meet this overwhelming need, and many residents still struggled for basic needs [9,10]. Many food-insecure households in New York City further experienced instability and insecurity in their jobs and health care at the same time.

It can be difficult to qualitatively analyze the experiences and opinions of individuals struggling with food access issues contemporary to the experiences themselves. However, social media (Twitter [subsequently rebranded as X] specifically) presents an opportunity to gather large amounts of real-time data from its users, as it can be used as a means of instant communication and information sharing. This allows researchers to obtain large amounts of data and insight into the opinions held by individuals during a discrete period. According to a survey of Twitter users conducted by Pew Research Center in 2018, individuals of all races and genders use the platform; however, users tend to be “younger, more educated and more likely to be Democrats than general public” [11]. Furthermore, Pew noted that 80% of all tweets originate from only 10% of all users, as the median Twitter user posts only about twice per month [11]. Previous research has shown that Twitter is frequently used to share and disseminate information to followers, while acknowledging the potential for the platform to be used to solicit information and assistance [12,13].

Numerous studies have attempted to track the posts made on Twitter that occurred during the COVID-19 pandemic, and some researchers have determined that social media could be a useful tool to observe and analyze the knowledge and opinions held by people during a public health emergency [14-16]. However, there is minimal social media–related research specific to food-related topics, presenting an opportunity to understand the barriers people face accessing affordable, nutritious, culturally appropriate foods and point those in need toward resources available to them [17,18]. Social media monitoring could be a useful asset to specifically understand the food environments and needs of individuals and families during such a time and potentially connect these people with resources available in their communities in real time.

### Objective

This study aims to explore the food access discourse during the COVID-19 pandemic on Twitter in New York City by analyzing publicly available tweets posted from March 1, 2020, to March 31, 2021.

## Methods

### Data Collection

Tweet collection began with determining keywords to search for, which was informed by the literature [14,19,20]. Expert knowledge informed initial keywords to search using the social media observation platform Talkwalker; this platform generates word clouds and lists sample posts containing keywords. These outputs were reviewed to search for and identify additional keywords using a snowball method, adding or removing keywords to include in our search depending on how relevant the related outputs were. Key phrases were searched in both English and Spanish. (Talkwalker has a proprietary artificial intelligence model used to determine a post’s language.) Slang keywords were added where applicable. For example, the term *freedge*, a portmanteau of *free* and *fridge*, was added to our list of search terms when use of this term was discovered in conversations about community fridges. Some terms were removed when it was determined that they would return too many irrelevant tweets. For example, in searching for tweets that talked about SNAP, called “SNAP” (uppercase), the term “snap” (lowercase) was removed after reviewing a sample of 100 tweets containing “snap” (lowercase) and determining none of them referred to SNAP.

These preliminary searches eventually resulted in lists of multiple keywords that were categorized into related subgroups, as informed by Kim et al [19]. These related subgroups are called *search strings* because similar key phrases were strung together with Boolean operators when conducting the search and collecting data using Talkwalker. A list of the search strings and the Boolean operators used to conduct these searches can be found in Multimedia Appendix 1. When searching using social media, there must be a balance between 2 criteria: “retrieval precision (how much of retrieved data are relevant) and retrieval recall (how much of the relevant data are retrieved),” as noted by Kim et al [19]. For the purposes of initial searches, retrieval recall was prioritized. Therefore, more tweets (and consequently more irrelevant tweets) were collected, and irrelevant tweets (also called *noise*) were removed during the data cleaning process. Tweets collected were posted between March 1, 2020, and March 31, 2021.

### Data Cleaning

Before analysis could occur, the data had to be cleaned to ensure all tweets were relevant to the study purpose. Tweets were deemed relevant to the study if they related to food insecurity and food access in New York City. Talkwalker has a multitiered process to determine the geolocation of tweets. First, Talkwalker will check their own internal database of users and authors and their locations. If the user is not listed there, they try to obtain location data from Twitter application programming interface if provided in the form of geographical coordinates. If these 2 steps yield no results, then it will check the user’s biographical location data. Finally, if still no location data can be found, then it will use other context clues (such as the username or the language) and place the tweet in the most likely country of origin’s capital (Talkwalker Product Support Representative, email, August 2024).

At least 50% of the time spent on this study was used to clean the data. Spam, porn, and irrelevant tweets were removed in a rigorous data cleaning process. Talkwalker has a feature that detects and categorizes spam tweets based on the type of spam the tweet is; any tweets categorized as “Social Media Account Promotions,” “Promotions,” “Job Offers,” and “Real Estate” were removed. Talkwalker has another feature that rates the “porn level” of any given tweet; tweets with a “porn level” higher than 0 were removed after reviewing a random sample of tweets with porn levels higher than 0 and finding none of the tweets to be relevant. Furthermore, posts with a porn level higher than 0 made up a negligible amount of less than 2%.

The data cleaning process included filtering out tweets that contained certain key phrases and hashtags not associated with food access and food insecurity. For example, in searching for tweets that talked about the SNAP, many tweets were retrieved about Snapchat (a social media company whose stock exchange ticker symbol is $SNAP), so all tweets that contained exactly the key phrases *$SNAP* and *Snapchat* were removed. The list of key phrases to remove was determined through a systematic review of a random sample of 100 tweets and adding irrelevant key phrases to a filter that removed them. A random sample of 100 tweets was continually generated and reviewed until at least 90% of the tweets in the sample were considered relevant to the purposes of the study.

**Figure 1:**
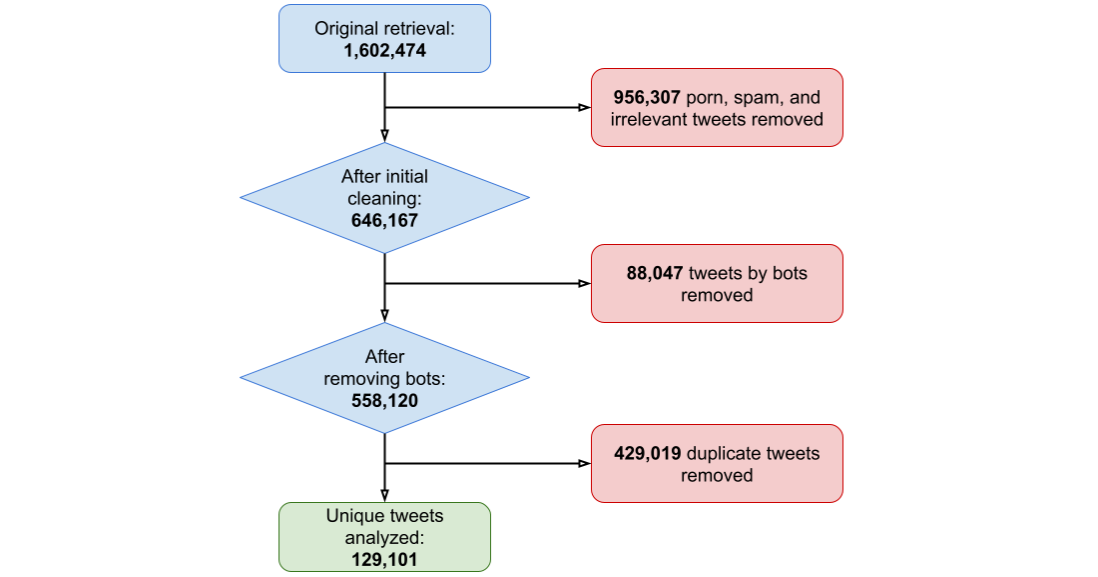
Flowchart of dataset cleaning process for collected tweets dated March 1, 2020, through March 31, 2021, in New York City.

During the data cleaning process, the search strings “Healthy Foods (En) & (Sp)” and “Fast Food” from the analysis were removed after random samples of the retrieved tweets were determined to be irrelevant to the purpose of the study. Furthermore, tweets retrieved by the “Community Fridges” and “Community Gardens” search strings were grouped together for analysis into one category, called “community efforts,” because these datasets comprised tweets with similar content. Finally, 3 search strings were “reverse filtered”: tweets that did not contain specific key phrases were removed. “Food Justice” was reverse filtered to only include tweets that contained at least one of the following phrases: *food*, *nutrition*, *meal*, *meals*, *nutritional*, *diet*, or *dietary*. “School Food” was reverse filtered to only include tweets that contained at least one of the following phrases: *food*, *nutrition*, *meal*, *meals*, *nutritional*, *diet*, *dietary*, *lunch*, *lunches*, *breakfast*, *breakfasts*, *eat*, *feed*, *feeds*, or *eats*. Finally, “Food Insecurity” was reverse filtered to only include tweets that contained the phrase *food insecurity*. Figure 1 depicts the number of tweets retrieved by the initial search, the number of tweets remaining after data cleaning, and the number of unique tweets that were analyzed.

Furthermore, tweets generated by bots (“partially or fully automated accounts on social media platforms,” according to Martini et al [21]) were removed using an application programming interface called Botometer. Botometer is a tool that “checks the activity of a Twitter account and gives it a score based on the extent to which it matches accounts that use automation” [22]. In other words, Botometer provides a score to a Twitter account based on how likely it is that the account is a bot; higher scores indicate a higher likelihood that the account is automated and not run by a human. Botometer was chosen to help determine which accounts were bots or not because it is the predominant tool used in the literature [11,23,24].

Botometer was used to score every Twitter account attached to the retrieved tweets (>600,000 accounts). Because Botometer does not decide whether a given account is a bot, instead only providing a score as to the likelihood that the account is a bot, a cutoff score to sort bots from nonbots had to be determined. Botometer provides 2 scores to each account checked: *raw* and *complete automation probability*. The raw score is the score that Botometer has assigned in terms of likelihood that an account is a bot. Higher raw scores mean a higher likelihood that an account is a bot, and lower raw scores mean a lower likelihood that an account is a bot. However, Botometer notes that raw scores “don’t provide enough information by themselves to classify an account.” Therefore, the complete automation probability score can be used to ascertain the “probability...that an account with this score or greater is controlled by software, i.e., is a bot.” For the purposes of this report, score refers to the raw score provided by Botometer. Various methods were considered to determine the Botometer cutoff score. A 2018 paper from the Pew Research Center analyzing bots used a Botometer cutoff score of 0.43 [11]. In addition, 3 research assistants were given a random sample of 150 accounts to decide “bot or not” based on attributes such as profile appearance, quantity of tweets and retweets, frequency of posting, and interactions with other users [25]. Interrater reliability was measured using Light’s implementation of the κ coefficient [26]. Furthermore, Amazon Mechanical Turk (MTurk) was used to crowdsource bot distinction for a random sample of 1366 accounts. However, due to various limitations to using MTurk (such as the inability to control for variation in graders by language proficiency or establish a shared definition for what qualifies as a bot) [27-29], ultimately the MTurk decisions were discarded and the research assistants’ determinations were used to test the Botometer thresholds. After exploration of both the research assistants’ cutoff score and the Pew Research Center paper cutoff score, and taking into consideration the performance metrics of specificity, sensitivity, accuracy, precision, and recall, a cutoff score of 0.43 was used to eliminate bot accounts, which removed approximately 11% of accounts.

Estimations of how many accounts on Twitter are bots were considered to justify the cutoff point. Each year, Twitter files a report with the US Securities and Exchange Commission noting how many active accounts on the website it considers to be automated, alleging about 5% of accounts are bots [30]. However, many researchers believe a more accurate estimate is 9% to 20% of all accounts [25,31]. When research assistants manually annotated accounts, they determined that approximately 11% of accounts were bots, roughly aligning with the literature.

After removing irrelevant tweets, tweets by bots, and duplicate tweets (refer to the Topic Modeling Analysis section for reasoning regarding the removal of duplicate tweets), 8.06% of our initial retrieval remained. As depicted in Figure 1, the search strings initially retrieved 1,602,474 tweets. Of these tweets, 956,307 tweets classified as porn, spam, or irrelevant to the study were removed; 88,047 tweets authored by bots were removed; and 429,019 duplicate tweets were removed. This left 129,101 unique, relevant tweets in the dataset for analysis.

Before topic modeling could occur, the tweets had to be further cleaned to optimize the results. Contractions (such as *I’m*, *it’s*, and *we’re*) were expanded (such as *I am*, *it is*, and *we are*). Punctuation and “stop words”—very common words in a particular language with little semantic value, such as *a*, *of*, and *to* [32]—were removed. The full list of stop words removed came from Natural Language Toolkit, the leading open-source natural language processing platform [33]. Emojis, hashtags, and tagged usernames (preceded by the @ symbol) were not removed.

### Topic Modeling Analysis

Topic modeling is a type of machine learning technique commonly used to evaluate data coming from a variety of text sources or documents to determine the words and features that the text sources or documents share [34,35]. A topic model scans a set of text sources or documents and groups them into *clusters* (sometimes called *topics*) based on shared words and phrases (called *keywords*).

A topic modeling technique called Gibbs Sampling Dirichlet Multinomial Mixture (GSDMM) was used because it is optimal for short texts and has been used in the literature [15,16,36-38]. Specifically, a GSDMM topic model publicly available on the software development sharing platform GitHub was used [39].

In addition, after much discussion and consideration, duplicate tweets and retweets from the topic modeling analysis were removed from the analysis. This resulted in a dataset wherein every tweet is distinct. This decision was made after some preliminary practice topic modeling was run and resulted in clusters entirely made up of a single tweet that had been retweeted several times. To better see the connections between the various *distinct* tweets, these retweets were removed. This left behind only one copy of each tweet in the dataset.

GSDMM topic modeling was then run on the dataset with the following parameters: *α* and *ꞵ* (parameters that affect the likeliness of tweets to sort into different clusters) set at .1, as recommended by Yin and Wang [37]. The number of iterations was set to 40, as Yin and Wang [37] noted that GSDMM does not require a high number of iterations for the model to reach convergence (ie, for the model to sort tweets into clusters with a high level of confidence). Each iteration is slightly different and more refined than the previous; iteration 40 is much more specific than iteration 1, but not significantly better than iteration 39.

Running GSDMM topic modeling resulted in a model unique to the dataset. This model revealed 50 clusters and their associated keywords. However, this model did not indicate the cluster of each individual tweet in the dataset, so then the model was used again on each tweet individually, producing a list of probabilities of that tweet belonging in each cluster. The tweet was then assigned to the cluster with the highest probability. The code used in the implementation of data cleaning and topic modeling can be found in a designated GitHub repository [40].

A qualitative content analysis of the clusters was then conducted. Informed by Ridhwan and Hargreaves [15], clusters that contained similar tweets and conversations were grouped into themes. This resulted in the emergence of 6 major themes. Each cluster was assigned to a theme. Only the top 30 most populated clusters were analyzed. Clusters determined to not be relevant to the study’s purpose were not assigned to a theme. For example, there was a cluster composed entirely of tweets quoting the Langston Hughes poem “Harlem,” which contains the line “Does it stink like rotten meat?” [41]. This cluster was eliminated from analysis. Furthermore, none of the clusters analyzed had any Spanish keywords, and the tweets analyzed were almost exclusively in English. After sorting the relevant clusters into their respective themes, the tweets were reviewed again to pull out significant subthemes within each major theme.

### Viral Tweets Analysis

While removing retweets was essential in ensuring the topic modeling clusters were not subsumed by viral tweets, excluding retweets from analysis altogether seemed like it would not lead to an accurate reflection of the conversations about food security and food access that took place on the platform. Thus, the content of and conversations instigated by certain viral tweets in New York City were analyzed. To do this, the top 10 most duplicated tweets (ie, the tweets with the most retweets occurring in New York City) were pulled from the dataset before retweets were removed. The replies and quote tweets posted on each viral tweet were then analyzed. Similar to the analysis of the clusters in topic modeling, in reviewing the replies and quote tweets, certain themes that characterized the conversations spurred by these tweets emerged.

### Interesting Terminology

During the review of the dataset, certain noteworthy terms and phrases were recognized. This included slang terms and words used in a nontraditional sense. These terms were pulled and investigated further for their use and potential implications on future social media research.

### Ethical Considerations

This study involved observations of large swaths of publicly available data, generally not considered to be “human subjects research.” Approval from an institutional research board was not needed. The proposed research did not require CUNY HRPP (City University of New York Human Research Protection Program) or Institutional Review Board review as we were not engaged in research involving human subjects, in accordance with CUNY guidelines [42]. All data were anonymized before analysis and publication. No compensation was provided to the individuals who wrote the tweets that were analyzed.

## Results

### Topic Modeling Analysis

Multimedia Appendix 2 shows the 6 themes and related subthemes that emerged from GSDMM modeling. Further information for each theme is also included, such as common words or roots and example tweets.

**Figure 2:**
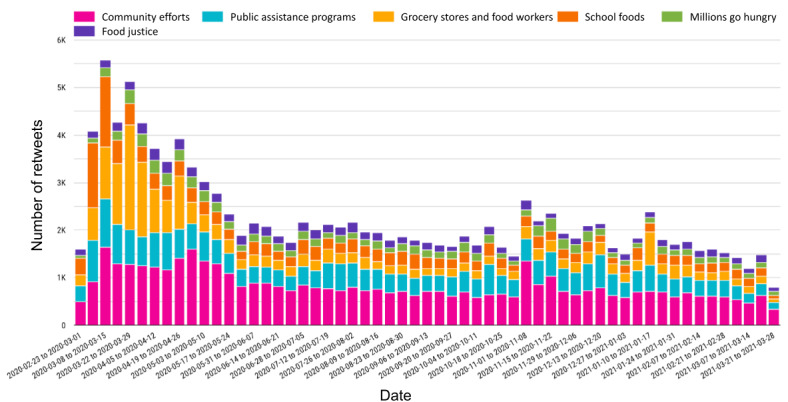
Distribution of tweets per theme by week from March 1, 2020, to March 31, 2021, shown by the relative number of tweets posted that fall within each theme.

Figure 2 shows the spread of tweets over the 13-month study period and the relative quantities of tweets in each of the 6 themes. Across all themes, there was a large spike in tweets in March and April 2020, which began to taper off during May 2020. The quantity of tweets stabilized from June through October 2020, experiencing relatively smaller spikes in November and December 2020, corresponding with the increased number of COVID-19 infections during this period.

Overall, the theme containing the most tweets was “community efforts,” as noted in Multimedia Appendix 2. This theme contained tweets related to community-based organizations providing assistance and relief to New Yorkers in need. As shown in Figure 2, the quantity of tweets related to this theme increased quickly in March 2020 and then began to taper off toward the end of May 2020. During this period, community efforts (such as community fridges, mutual aid organizations, and other nonprofits) quickly scaled up to meet the needs of New Yorkers. Another spike in tweets related to this theme occurred in the middle of November 2020, ahead of the holiday season and amid increased COVID-19 infection rates.

The second most populated theme was “public assistance programs,” which included any tweets related to programs such as SNAP and pandemic electronic benefit transfer (P-EBT). Figure 2 shows that these conversations increased quickly in the early months of the pandemic, when SNAP benefits were temporarily increased for all recipients after passage by the US Congress of the Coronavirus Aid, Relief, and Economic Security Act in April 2020 [43], but tweets tapered off by June 2020. Minor spikes can be seen in July 2020, when the P-EBT was expanded to all public school students in New York City [44], and in December 2020, during the holiday season. Twitter users also worried about electronic benefit transfer (EBT) cards not having sufficient funds to purchase food, stores being unable to process EBT transactions, and the purchasing decisions made by people receiving SNAP benefits.

The third most populated theme was “grocery stores and food workers.” Tweets belonging to this theme pertained to shopping habits during the pandemic and protections for people working in the food industry during the pandemic. Some users posted about the benefit of web-based grocery delivery services (such as Instacart and Amazon Fresh) accepting SNAP [45,46]. This also included a very small number of tweets regarding the safety of products at the grocery store and a misinformation theory that certain packaged frozen foods were contaminated with COVID-19. This theme saw the highest number of tweets in April and May 2020, as there was a nationwide strike by grocery store and other food workers on May 1, 2020. Furthermore, this theme saw a smaller spike in January 2021, when Teamsters at the Hunts Point Produce Market went on strike [47].

The fourth most populated theme was “school foods.” This theme contains tweets about how students were going to access meals without in-person schooling and discussions about P-EBT or grab-and-go meals. This theme saw a large spike in March 2020, when schools were closed in New York City, and smaller spikes in July 2020 and September 2020, other moments of uncertainty about students’ futures.

The fifth theme with the most tweets was titled “millions go hungry.” This theme is characterized by tweets containing information about the pandemic’s effects on food insecurity but on a broader scale (ie, not specific to New York City). Users commonly tweeted about the “millions” of households that will experience food insecurity because of the COVID-19 pandemic, drawing attention to the inadequacy of existing infrastructures (such as food pantries, benefit programs, and charities) to meet the expanded demand. This theme was fairly consistent throughout the period analyzed.

The sixth and final theme that emerged from the data was “food justice.” Tweets in this theme related to the relationship between social justice or progressive ideology and food. This theme saw spikes from May through August 2020, in the wake of nationwide Black Lives Matter protests spurred by the murder of George Floyd. This theme also saw a smaller spike in November 2020, during the days leading up to the November 3, 2020, federal election.

### Analysis of Viral Tweets

Multimedia Appendix 3 shows the top 10 viral tweets pulled from the dataset along with the number of likes and retweets they each received, as well as a brief summary of the types of conversations found in the replies to these tweets. Top viral tweets received 6946 to 68,784 retweets and 24,794 to 298,839 likes. Most tweets came from users with follower counts in the thousands to millions, but 1 viral tweet about a major worker strike in the food industry was posted by a user with only 956 followers at the time.

**Figure 3:**
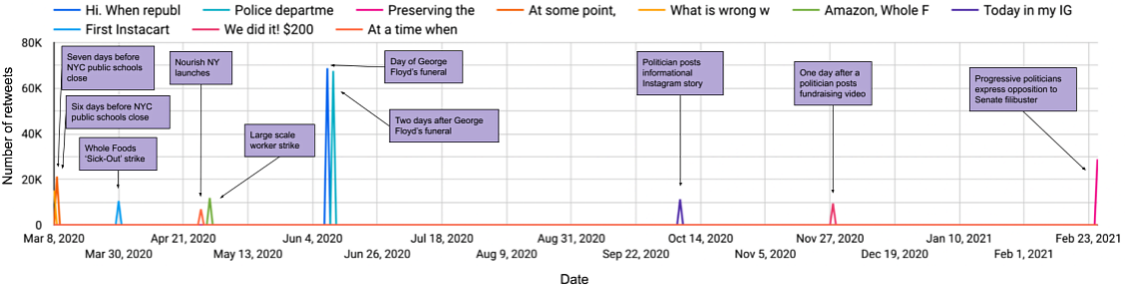
Viral tweets over time by number of retweets, with added context about current events occurring at similar times to the tweets. NY: New York; NYC: New York City.

Figure 3 shows the top 10 viral tweets over time, mapped to the timelines when they went viral. The content of these tweets was analyzed relative to contemporary news and current events.

The first 2 tweets to go viral in the dataset occurred in early March 2020, a few days before Mayor Bill de Blasio announced the closure of New York City public schools as a precaution against COVID-19 [48]. Conversations instigated by these tweets primarily related to students from low-income families and concerns that they might not be able to have reliable meals without in-person schooling.

Two more top viral tweets related to large-scale food worker strikes that occurred during the early months of the pandemic. On March 31, 2020, workers at Whole Foods staged a nationwide “sick-out” strike (a strike wherein employees call out from work sick) to demand COVID-19 precautions [49]. A little over a month later, on May 1, 2020, workers for Amazon, Instacart, Whole Foods, and others staged a strike to protest the lack of protections and benefits to frontline workers [50].

Many viral tweets were by or about politicians and related to food access and food policy. Tweets of this kind instigated conversations about individuals’ political leanings and support. Furthermore, these tweets tended to garner more disagreement within the replies and quote tweets. One tweet by the same politician referred to a push by Democrats in February 2021 to eliminate the Senate filibuster [51]. Another referred to the passing of a state initiative to reroute surplus agricultural output to food banks in the state, called Nourish New York.

Another tweet by a politician related to the Black Panther Breakfast Program [52], which led to discussions about free and reduced-price school meals in the 21st century and the relationship between food and racial justice. Two viral tweets occurred during the early June 2020 Black Lives Matter protests, especially in the days surrounding George Floyd’s funeral. These tweets prompted conversations about police brutality, one of which directly compared police budgets to school budgets and the affordability of school food.

### Interesting Terminology

In reviewing the tweets, certain interesting terms emerged from the data that could help guide social media studies and interventions in the future.

- “School lunch debt” is a phenomenon wherein students who are unable to pay for their daily school lunches and accrue a balance they owe to the school. Some schools would cease to provide lunch to students whose “lunch debts” were too high; example tweet: “Please explain f—school lunch debt. Why are 7 year olds in debt over chicken nuggets.”
- A “struggle meal” refers to a meal that someone would eat during times when money is tight; example tweet: “Facts, my struggle meal was one slice of bread with butter. Or if I was lucky chopped hot dogs w. Ketchup. And now that’s basically my snack.”
- People in need of food assistance sometimes turned to Twitter to ask other users for help, usually through gifts of cash. These types of posts varied quite a bit in the language used, but almost all of them included a link to a CashApp, PayPal, or Venmo account; example tweet: “The only thing that could possibly be good about this Xmas would be if you see my entering 1000x for cashapp blessings and helping me so I don't lose my home. [username] Unemployed, no money, can't pay rent, get food, will be homeless. Been in survival mode too long. Breaking.”
- With the proliferation of community fridges during the pandemic came new terminology related to them, including the word “freedge”—a portmanteau of “free” and “fridge”; example tweet: “In my neighborhood in NYC there is a Freedge—a free refrigerator where we can all put food for others to take as they wish.”
- In the context of government assistance, critics of the support used the term “freeloading” often to point toward people who were taking advantage of the benefits for a long time or without an effort to get off them; example tweet: “I know so many women that have been freeloading off government assistance and bragging about their benefits for years.”

## Discussion

### Principal Findings

This study analyzed tweets during the first 13 months of the COVID-19 pandemic related to food security and food access in New York City. The themes that emerged from the data include topics related to food justice, food policy, food workers, and food relief efforts. Overall, quantities of tweets increased in correlation with current events, such as the closure of New York City public schools [53]; the expansion of SNAP and unemployment benefits [43,54]; the proliferation of mutual aid groups in April and June of 2020 [55]; and the May Day Instacart, Amazon, and Target strike in 2020 [56]. The study highlighted some of the common opinions held, emotions felt, and phrases used by New Yorkers from March 2020 to March 2021 around these issues.

In the earliest months of the pandemic (March, April, and May 2020), many tweets were posted about food access and community responses to the COVID-19 pandemic. This included changes in shopping habits and increased awareness of food resources such as SNAP benefits, food pantries, and community fridges. Due to stay-at-home orders and worries about catching COVID-19 from shoppers at the grocery store, web-based grocery shopping became increasingly popular during this time [57,58]. However, with this increased demand for web-based shopping came delayed grocery deliveries and errors in grocery orders, about which Twitter users frequently complained. Other users posted about the opening of new grocery stores in their communities and the expansion of grocery delivery areas.

During the early months of the pandemic, many individuals saw food shortages [59,60], but some users noted that Spanish, Mexican, and Chinese grocery stores had many products in stock while more mainstream grocery store chains had empty shelves. Larger food distributors, including large-scale meat processing plants and fruit and vegetable production facilities [61,62], were especially susceptible to pandemic-caused closures and service interruptions [63,64]. It is possible that some of these stores rely on more direct supply chains that were not as heavily impacted by pandemic closures, thus allowing the store to continue purchasing inventory directly from farmers and producers without experiencing as many interruptions as larger grocery store chains [65,66]. Furthermore, another possible reason that smaller ethnic grocery stores were more likely to have products on the shelves than larger supermarkets is due to the stockpiling that many individuals participated in during the early months of the pandemic [67]. Someone interested in stockpiling large amounts of product is less likely to shop at a small ethnic market, rather they would go to a large, multidepartment store that carries a larger variety of products.

Government officials also became involved in Twitter conversations, sharing information about new policies and resources available to New Yorkers. For example, in the early months of the pandemic, dairy farms were forced to dump old milk as the lockdown restricted the market for dairy products [68]. In response to this, the Governor announced a new policy to combat food waste and food shortages simultaneously, called Nourish New York [69]. The Nourish New York initiative “reroutes surplus agricultural products to populations who need them most through NY’s network of food banks,” according to the New York State Department of Agriculture and Markets website [69]. Farmers and other food producers in New York State share information about surplus food products and are connected to food banks that purchase these items to distribute to people in need. The initiative was highly successful and has since become permanent [70]. The supply chain disruptions and food shortages caused by the pandemic, especially in large-scale plants and facilities [63,64], showed a need to strengthen regional food chains to create a more resilient system [63].

Conversations were often politicized: some users talked about fears that Republican politicians sought to cut SNAP budgets or make enrolling in the program more difficult. SNAP benefits are already often insufficient to meet a household’s full food needs [71-73]. In fact, toward the end of each month, food pantries have reported increased need from clients, many of whose SNAP benefits have likely run out [74]. This phenomenon was also seen in the data: some Twitter users in need posted about running out of money on their EBT cards and needing assistance toward the end of the month, asking others who saw the posts to help them out with gifts of cash. The need for extra money to purchase food has persisted beyond the first year of the pandemic; inflation [75] and the end of expanded SNAP benefits in 2023 [76] have shown continual barriers for people needing assistance acquiring food. There are clear implications on potential policy of these obstacles to meeting basic needs faced by many of the country’s most susceptible populations.

There were also numerous debates about what kinds of foods SNAP beneficiaries should be allowed to purchase with their benefits; in November 2020, a celebrity posted a tweet about using EBT cards to purchase only healthy items, drawing criticism from users who wrote that no one should be allowed to police what others eat. Currently, SNAP recipients can purchase any food items for the household, such as fruits, vegetables, meats, dairy, grains, and “accessory foods” [77]—this includes cooking supplements such as oil, baking powder, and sugar, as well as snacks, desserts, and packaged beverages [78]. However, hot food items are generally not SNAP eligible. In 2021, the Restaurant Meals Program, a state option to allow certain populations to purchase hot food with SNAP benefits, was passed [79]; as of publication, the program is not available in all parts of New York State and is in the process of rolling out in New York City. Municipalities not opting into this program can leave many unhoused, disabled, and older adult New Yorkers who are unable to prepare or cook food with another barrier to accessing nutritious, warm meals.

Negative perception of the choices made by SNAP beneficiaries regarding what foods they choose to purchase using benefits is ubiquitous, resulting in a stigmatization of the program that often dissuades eligible people from enrolling [80,81]. It also feeds into the detrimental misconception that people receiving benefits are “freeloading,” one of the terms found in the data. However, these arguments that SNAP beneficiaries should not be allowed to purchase “junk food” or other *unhealthy* items fail to recognize the food environment of many people enrolled in the program. Many underresourced communities with higher rates of poverty have fewer full-service grocery stores and more bodegas than affluent communities, so individuals in these communities are less likely to shop at supermarkets with larger inventories of “healthy foods” [82-85]. Furthermore, *healthy foods* are often perceived as more expensive than *unhealthy foods*, another barrier to healthy eating [86-89]. Consider the term “struggle meal” as found in the data: many of these struggle meals were described as quite calorically dense and comprised processed or prepackaged foods. Lack of access to affordable healthy foods contributes to the higher rates of diet-related diseases, such as diabetes and obesity, seen in underresourced communities.

School foods and getting meals to children was a top concern for Twitter users, especially when public schools closed in March 2020, during the summer vacation of 2020, and when schools were supposed to reopen in September 2020. Many users lamented how for some students, their free school breakfast and lunch was the only guaranteed meal they had each day. The P-EBT program was significant in helping families with children to acquire food [90,91]. Because New York City has universal free school meals, all families with children enrolled in public school received preloaded P-EBT cards, regardless of need. Automatic enrollment in the P-EBT program proved to be an effective way to ensure that all children in need received the benefit and positive health impacts associated [92]. Furthermore, some well-off families expressed interest in donating their surplus P-EBT funds to others in need [93,94]. P-EBT is also a more cost-effective measure for school districts than grab-and-go meals, another program meant to replace the meals lost by pandemic school closures [95]. However, many schools faced administrative obstacles in implementing the P-EBT program, leaving many families without benefits for as much as half the school year [96].

Some users also mentioned a phenomenon called “school lunch debt,” wherein students are denied breakfast or lunch at school because they owe money for past lunches [97,98]. Some Twitter users, including 1 viral tweet with tens of thousands of likes and retweets, posted about the Black Panther Party’s Breakfast Program of the 1960s and 70s [99] as a reference point for free school meals. Meals for schoolchildren is a particularly salient topic because of the various harmful effects of food insecurity on children’s health and development [100-102]. Research has shown that food insecurity during childhood is associated with health, emotional, behavioral, and academic problems that might persist into adulthood [100-102]. Children from food-insecure households are also more likely to be suspended or expelled from school [103]; increased rates of suspension and expulsion, in turn, are associated with the school-to-prison pipeline [104,105]. Future research should be explored into the relationship between food insecurity in a community and the school-to-prison pipeline.

Viral tweet analysis offers an interesting look into what posts and ideas gained the most traction and public discourse. While tweets from ordinary users without large follower counts provide an important glimpse into the opinions and perspectives of residents during this time, viral tweets are, by their nature, seen by far more users and have the potential to contribute more heavily to the public understanding of the topics they discuss [106]. Therefore, users with large followings are better able to expose other Twitter users to important and underdiscussed topics including urban food insecurity, labor activism, and nutrition assistance programs.

There is often a gap between individuals in need of nutrition assistance and organizations, programs, and services that exist to provide this necessary assistance. Often, individuals most in need of help are unable to access the resources available within their communities, whether due to limited hours or availability, lack of awareness, or stigma [107-109]. Therefore, social media provides a unique platform to quickly connect individuals and families in need with support from their own neighborhoods, as well as normalize and destigmatize the use of these services. By combining location data with an understanding of keywords and terminology used by people in need, service providers have the potential to intervene and offer their services more quickly and efficiently to their target populations. Furthermore, by understanding what makes certain posts go viral, hunger activists and food relief service providers might better be able to spread awareness of their efforts within affected communities. In addition, given the range of the authors of the viral tweets—politicians, academics, labor union activists, nutritionists and personal trainers, political analysts, and fashion journalists—leveraging collaborations with influencers across various sectors could further enhance the reach, delivery, and impact of critical services.

### Limitations

While this study successfully analyzed tweets about food insecurity and food access in New York City during the COVID-19 pandemic, there were certain limitations. First, in cleaning the tweets of bots, it was learned that bot detection is not an exact science. Some tweets might have been flagged as bots even if the users were real, and some tweets by bots might have evaded detection and were kept in the dataset. Second, Talkwalker, the platform used to pull tweets, was not good at reading images, despite the initial study conceptualization to include an analysis of images.

Previous social media monitoring studies have demonstrated the prevalence of misinformation on platforms [110-112]. Therefore, it is possible that some tweets analyzed contained incorrect information. To better spread reliable information during a public health emergency such as the COVID-19 pandemic, social media is an invaluable tool for policy makers to understand the way public health information is shared. Future social media monitoring studies should consider and analyze any misinformation found within the dataset.

Finally, for the analysis of viral tweets, the number of likes and retweets each tweet accumulated was looked at. However, these numbers reflect these metrics from when the data were collected in Winter 2022. Therefore, it is possible that by the end of the time analyzed, March 31, 2021, these figures were lower and that more likes and retweets were added in the 7 months before the data were collected. However, it is unlikely that this altered the figures significantly. This is because as time goes on, newer tweets push these viral tweets down on the home page, and users are therefore less likely to see them [113].

This study only looked at social media posts on Twitter, but conversations on other platforms, such as Facebook and Instagram, could be beneficial to monitor in a similar way. Analyzing images posted on social media in addition to text would also be beneficial. The quantity of tweets in Spanish was so low that most Spanish tweets were ignored by topic modeling software. Future research should separate Spanish and English tweets before topic modeling to avoid this issue. Furthermore, it is possible that Spanish-speaking New York City residents do not use Twitter at the same rates as English-speaking residents, or they may use Twitter in English and other platforms in Spanish; therefore, future research should hone in on social media platforms used more prominently by Spanish speakers.

Since this study was originally conducted, Twitter was purchased and rebranded as a new social media platform called X. This raises questions about the reproducibility of the study now that Twitter, as it was once known, no longer exists. Talkwalker representatives stated that all data and accounts from Twitter were not deleted or otherwise removed in the change to X (Talkwalker Product Support Representative, email, August 2024). Any tweets no longer in existence on X are a result of the user’s decision to remove them. Therefore, any researcher who would like to retrieve the same data used in this study would need only the same Talkwalker access and Boolean search strings used here. However, the General Data Protection Regulation (GDPR), implemented in the European Union in 2018, requires companies to delete any stored digital data if a user requests so [114,115]. Therefore, if a user deletes a post or account from X, Talkwalker is also required to delete any data they have associated with that account or post. The GDPR only affects companies operating in the European Union, and because Talkwalker is a global company, this policy does apply to them. Talkwalker also chooses to abide by these standards for users and posts located outside of the European Union (Talkwalker Product Support Representative, email, August 2024). While it is unknown exactly why Talkwalker chooses to do so, there are several reasons a company may do this. One potential explanation is that the GDPR can be seen as the highest standard for digital rights, and by adhering to it in countries with different standards, they are not only meeting the requirements of the host country but also providing protections above and beyond that.

It is unclear what the full impact of the change from Twitter to X will be on future social media listening research, including studies such as this one. Since the rebrand to X, many users migrated away from the platform and onto other social media websites, including sites such as Bluesky, Threads, and Mastodon [116]. Some analysts estimated that Twitter lost as many as 1 million users in the months after the company’s ownership change that will eventually lead to the rebranding to X [117]. However, many of these alternative sites were far less successful than Twitter, and some users eventually migrated back to X. Many users have concluded that the transition from Twitter to X had little significant impact on the experience of using the social media platform [116,118].

In sum, if any accounts or posts were deleted since data for this study were initially retrieved, they would be unavailable to future researchers. This is not a new phenomenon. For many studies involving computational communication science, as more time passes from the data collection stage of a study, less and less data are available to future researchers as a result of account and post deletions, changed privacy settings, and user suspensions [119].

### Conclusions

Findings reveal that in the earliest months of the COVID-19 pandemic, Twitter users in New York City quickly responded to the wave of need by sharing information and resources about food access in their communities. Some users turned to Twitter to either solicit or offer help finding food. Furthermore, the platform lent itself to many conversations about the policies enacted on a federal, state, and city level to help feed New Yorkers in need. Future research on this topic should include an analysis of social media posting on platforms such as Facebook, TikTok, and Instagram, as well as in languages other than English. Social media data can be used to explore and better understand the food security and food access challenges and concerns of local communities and how such challenges and concerns may evolve over the course of the pandemic. Community leaders and elected officials could use such data to inform more responsive and tailored initiatives and services to better address such crises.

## Appendix

Multimedia Appendix 1 Boolean search strings.

Multimedia Appendix 2 Themes and related subthemes from topic modeling analysis of collected tweets, presented with the common words or roots, the percentage of tweets from the dataset that fall into each theme, and at least 1 anonymized example tweet per subtheme (N=129,101).

Multimedia Appendix 3 Top 10 viral tweets, in chronological order of original post, presented with each tweet’s total number of likes and retweets at the time of data collection and a brief qualitative summary of the content posted in replies and quote tweets.

## Abbreviations

EBT: electronic benefit transfer
GDPR: General Data Protection Regulation
GSDMM: Gibbs Sampling Dirichlet Multinomial Mixture
MTurk: Mechanical Turk
P-EBT: pandemic electronic benefit transfer
SNAP: Supplemental Nutrition Assistance Program
