# Supplementary material for: Food Access in New York City During the COVID-19 Pandemic: Social Media Monitoring Study
Source: JMIR Form Res. 2025 May 9;9:e49520. doi: 10.2196/49520 (PMC12102621; doi:10.2196/49520)
Supplement: Multimedia Appendix 1 [file formative_v9i1e49520_app1.docx]

Multimedia Appendix 1: Boolean search strings

1. EBT/SNAP: (“ebt” OR “food stamps” OR ++”SNAP” OR “snap benefits” OR “p-ebt” OR “fmnp” OR “health bucks” OR ((“cupon*” OR “alimento*” OR “ebt” OR “snap”) NEAR/5 “beneficio*”)) NOT “descuento*”
2. WIC/eWIC: ((“wic” OR “ewic” OR “fmnp”) NEAR/5 (“food” OR “eat*” OR “feed*” OR “health*” OR “salud” OR “sano” OR “farmers market” OR "mercado de agricultores" OR "mercado sobre ruedas" OR “comida”))
3. School Food: (((“school” OR “escuela*” OR “escolar”) AND (“meal*” OR “lunch*” OR “food” OR “breakfast*” OR “free” OR “comida” OR “desayuno*” OR “almuerzo” OR “gratis” OR “descuento”)) OR (“reduced” AND (“meal*” OR “lunch*”)))
4. Food Prices: (“food” OR “comida” OR “supermarket*” OR “supermercado*” OR “bodega*” OR “grocery” OR “abaceria” OR “abbarroteria” ) NEAR/5 (“cheap” OR “barat*” OR “expensive” OR “car*” OR “broke” OR “skint” OR “too much” OR “coupon” OR “cupon*” OR “sale” OR “descuento”)
5. Grocery Stores/Brands: (“food bazaar” OR “aldi” OR “key food*” OR “c town” OR “target grocer*” OR “western beef” OR “meat market” OR “shoprite” OR “trade fair” OR “stop n shop” OR “foodtown” OR “super duro” OR “instacart” OR “freshdirect” OR “shipt” OR “peapod”)
6. Fast Food: (“mcdonald*” OR “burger king” OR “kfc” OR “white castle” OR “boston market” OR “popeye*” OR “taco bell” OR “chipotle”)
7. Community Gardens: (“garden*” OR “farm*” OR “jardin*” OR “huerto”) NEAR/5 (“commun*” OR “urban” OR “pantry” OR “mutual aid” OR “comunitario” OR “comunidad”)
8. Community Fridges: (((“fridge” OR “refrigera*” OR “nevera*”) NEAR/5 (“commun*” OR “free” OR “mutual aid” OR “comunitari*”)) OR “freedge*”)
9. Food Pantries: (“pantr*” OR “kitchen” OR “distribut*” OR “despensa*” OR “canasta*”) NEAR/5 (“food” OR “soup” OR “free” OR “comida” OR “alimento*” OR “gratis” OR “entrega”)
10. Food Insecurity: (“food insecurity” OR “food security” OR “inseguridad aliment*” OR “hungry” OR “hambre” OR “hambrient*” OR “starving” OR “enough food” OR “suficiente comida”)
11. Struggle (Eng): (“struggl*” OR “hard time*” OR “unemploy*” OR “homeless” OR “lost job”) NEAR/5 (“food” OR “meal” OR “feed” OR “hungry”)
12. Food Justice (Eng): (“food” OR “healthy food” OR “health” OR “diet”) NEAR/5 (“justice” OR “apartheid” OR “equit*” OR “inequit*” OR “disparit*” OR “racis*”)
13. Food Quality (Eng): (“produce” OR “food” OR “fruit*” OR “vegetable*” OR “veggies” OR “meat*”) NEAR/5 (“gross” OR “nice” OR “quality” OR “rotten” OR “moldy” OR “expired” OR “fresh” OR “look* good”)
14. Access to Culturally Appropriate Foods (Eng): (“kosher” OR “halal” OR “caribbean” OR “chinese” OR “indian” OR “dominican” OR “jamaican” OR “mexican” OR “puerto rican” OR “spanish” OR “ground provisions”) NEAR/5 (“food pantr*” OR “food bank*” OR “grocer*” OR “supermarket*” OR “soup kitchen*” OR “food box” OR “community fridge*” OR “mobile pantry”)
15. Healthy Foods (Eng): (“nutritious” OR ((“unhealthy” OR “healthy”) NEAR/3 (“food*” OR “eat*” OR “restaurant*”)) OR (“food” NEAR/3 (“good for you” OR “bad for you”)))
16. Struggle/Lucha (Sp): (“lucha” OR “tiempo* dificil*” OR “desemplead*” OR “sin hogar” OR “sin trabajo”) NEAR/5 (“comida” OR “alimento*” OR “alimentar”)
17. Food Justice (Sp): (“comida” OR “comida saludable” OR “saludable” OR “dieta”) NEAR/5 (“justicia” OR “equidad” OR “desigualdad” OR “disparidad” OR “racismo”)
18. Food Quality (Sp): (“verduras” OR “vegetales” OR “frutas” OR “carne” OR “comida”) NEAR/5 (“de calidad” OR “descompuesto*” OR “podrid*” OR “caduc*” OR “fresco”)
19. Access to Culturally Appropriate Foods (Sp): (“caribeno” OR “dominicano” OR “puerto riqueno” OR “latin*” OR “mexican*” OR “spanish” OR “salvadoreno” OR “colombiano” OR “venezolano” OR “peruano” OR “ecuatorian*”) NEAR/5 (“banco de alimentos” OR “supermercado” OR “comedor de beneficiencia” OR “caja de alimento*” OR “refrigerador comunitario” OR “nevera comunitaria”)
20. Healthy Foods (Sp): (((“nutritiv*” OR “saludable” OR “no saludable”) NEAR/5 (“alimento” OR “comida” OR “restaurante”)) OR ((“comida” OR “alimento” NEAR/3 (“bueno para ti” OR “malo”))))
