# Supplementary material for: Food Access in New York City During the COVID-19 Pandemic: Social Media Monitoring Study
Source: JMIR Form Res. 2025 May 9;9:e49520. doi: 10.2196/49520 (PMC12102621; doi:10.2196/49520)
Supplement: Multimedia Appendix 2 [file formative_v9i1e49520_app2.docx]

**Multimedia Appendix 2.** Themes and related subthemes from topic modelling analysis of collected tweets.

| Themes and clusters | Subthemes | 10 most common words or roots | Tweets in dataset, n (%) | Example tweet |
| --- | --- | --- | --- | --- |
| Community efforts: 11, 26, 31, 34, 42, 24, 49, and 38 | 1. Announcements about program reach or impact, volunteers, donors, and partnering organizations 2. Information sharing about upcoming food distribution events 3. Discussions about community fridges (eg, efforts to keep them stocked and incidents of vandalism) 4. Discussions about where residents can acquire fresh foods (eg, community gardens and food distribution events) | - “food” - “fresh” - “distribut” - “pantri” - “communiti” - “help” - “like” - “donat” - “will” - “free” | 47,207 (36.57) | - “We are so thankful for this amazing contribution to our Halal Food Pantry. While Ramadan celebrations look a little different this year, donations like these help bring a sense of normalcy to those we serve in our community. #COPO #ramadan” - “Our doors are open today at the Times Square community center! If you’re struggling during this #COVID19 pandemic, visit us at 315 West 47th St. We’re here to serve anyone who is in need through our food pantry. We’ll see what we can do to help support you and your family. 🙏” - “Haven’t shared this in a while, but if you’re in NYC & don’t know where your nearest community fridge is, here’s a map. I expect many of us may be able to give to support these, so please do! (If you’re in other cities, share your maps, too. 🙏🏾💜)” - “If you need help supplementing your groceries with fresh produce, milk, whatever, see if your neighborhood has a community fridge” |
| Public assistance programs (eg, SNAP^a^): 1, 40, 36, and 5 | 1. Opinions for or against proposals and ideas for policy changes related to receiving benefits (eg, new work requirements and expanded eligibility) 2. Discussions regarding major policy changes to benefit programs (eg, P-EBT^b^ and online SNAP purchasing) 3. Direct requests for help to pay bills and buy food | - “food” - “peopl” - “stamp” - “snap” - “need” - “can” - “get” - “homeless” - “unemploy” - “will” | 26,446 (20.48) | - “The right to eat doesn’t mean you can just go to the grocery store and get what you want and not pay champ. We have food stamps that people can apply for. We already have 1 party worried that providing food & shelter disincentivizes work we need 1 that understand what empathy is.” - “One of the most effective ways we can protect families from hunger is to #boostSNAP (formerly known as food stamps) & help more Americans enroll in the #SNAP program. Call your senator now to ask for an increase in SNAP benefits in the next #reliefbill #HungerActionMonth” - “I’m so fucking hungry. I have two cans of chicken and rice soup and some protein powder, and that’s it. I get food stamps on the first. Please can anyone spare a couple dollars for food? PayPal [username] Venmo CashApp [username]” |
| Grocery shopping and food workers: 44, 8, and 27 | 1. Discussions regarding the safety of shopping in person for at-risk populations (eg, special supermarket hours for older adults and shopping outdoors at farmers’ markets) 2. Discussions regarding the ethics of grocery delivery (eg, fair pay, tipping etiquette, and striking app delivery workers) 3. Discussions about barriers to shopping (eg, online use of EBT^c^ and increased cost of fresh fruits and vegetables) | - “instacart” - “food” - “groceri” - “worker” - “deliveri” - “get” - “order” - “store” - “can” - “time” | 20,515 (15.89) | - “Aldi launches home delivery service for vulnerable and self-isolating customers” - “Please stand by workers today as they strike for basic protections, sensible paid leave policies, and hazard pay to account for the inherent danger of working on the frontlines in a pandemic. Don’t buy from Amazon, Target, Instacart, or Whole Foods.” - “Uggggggg..i can’t get a food delivery from anyone till after Easter. Now Instacart going on strike. So many people going to grocery stores. I would go, but can’t chance it. I take care of my 90 year old mom. If I come down with it or even just become carrier noooooo goooood” |
| School foods: 15 and 16 | 1. Information sharing and announcements regarding P-EBT and grab-and-go school meals programs 2. Discussions regarding feeding food-insecure students during the pandemic | - “school” - “lunch” - “meal” - “food” - “student” - “Need” - “children” - “kid” - “day” - “can” | 16,023 (12.41) | - “Attention public-school families! NYC families are being sent P-EBT cards, regardless of income. Every single public-school student will receive $420 in P-EBT. If you don’t need it, these funds can be shared with food insecure families.” - Retweet: “On a typical school day, 21 million #K12 students from low-income backgrounds rely on free or reduced-price breakfast & lunch. With schools across the country closed indefinitely the U.S. is facing the risk of massive child hunger.” |
| Millions go hungry: 37, 18, and 39 | 1. Discussions about the state of food insecurity and unemployment because of the COVID-19 pandemic | - “food” - “insecur” - “pandem” - “need” - “peopl” - “new” - “covid” - “help” - “million” - “health” | 9103 (7.05) | - “Heartbreaking that millions of U.S. families experience hunger and food insecurity, and not just during the pandemic. Food banks, pantries & charities are struggling to meet the demand. Let’s do what we can to help & not look away.” |
| Food justice: 2 and 4 | 1. Discussions about the impact of climate change on health and food sources, grassroots efforts in addressing food security, food justice, and food waste | - “food” - “communiti” - “fresh” - “garden” - “produc” - “can” - “farm” - “distribut” - “qualiti” - “need” | 9004 (6.97) | - “There are many people working on the grass-roots level to help mitigate these issues. Many Black people are working in urban and rural agriculture to provide fresh quality produce and foods to communities in need.” |

^a^SNAP: Supplemental Nutrition Assistance Program.

^b^P-EBT: pandemic electronic benefit transfer.

^c^EBT: electronic benefit transfer.
