# Supplementary material for: Food Access in New York City During the COVID-19 Pandemic: Social Media Monitoring Study
Source: JMIR Form Res. 2025 May 9;9:e49520. doi: 10.2196/49520 (PMC12102621; doi:10.2196/49520)
Supplement: Multimedia Appendix 3 [file formative_v9i1e49520_app3.docx]

**Multimedia Appendix 3**. Themes and related subthemes from topic modeling analysis of collected tweets, presented with the common words/roots, the percentage of tweets from the dataset that fall into each theme, and at least 1 anonymized example tweet per subtheme (N=129,101).

| Themes and clusters | Subthemes | 10 most common words or roots | Tweets in dataset, n (%) | Example tweet |
| --- | --- | --- | --- | --- |
| Community efforts: 11, 26, 31, 34, 42, 24, 49, and 38 | 1. Announcements about program reach or impact, volunteers, donors, and partnering organizations 2. Information sharing about upcoming food distribution events 3. Discussions about community fridges (eg, efforts to keep them stocked and incidents of vandalism) 4. Discussions about where residents can acquire fresh foods (eg, community gardens and food distribution events) | - “food” - “fresh” - “distribut” - “pantri” - “communiti” - “help” - “like” - “donat” - “will” - “free” | 47,207 (36.57) | - “We are so thankful for this amazing contribution to our Halal Food Pantry. While Ramadan celebrations look a little different this year, donations like these help bring a sense of normalcy to those we serve in our community. #COPO #ramadan” - “Our doors are open today at the Times Square community center! If you’re struggling during this #COVID19 pandemic, visit us at 315 West 47th St. We’re here to serve anyone who is in need through our food pantry. We’ll see what we can do to help support you and your family. 🙏” - “Haven’t shared this in a while, but if you’re in NYC & don’t know where your nearest community fridge is, here’s a map. I expect many of us may be able to give to support these, so please do! (If you’re in other cities, share your maps, too. 🙏🏾💜)” - “If you need help supplementing your groceries with fresh produce, milk, whatever, see if your neighborhood has a community fridge” |
| Public assistance programs (eg, SNAP^a^): 1, 40, 36, and 5 | 1. Opinions for or against proposals and ideas for policy changes related to receiving benefits (eg, new work requirements and expanded eligibility) 2. Discussions regarding major policy changes to benefit programs (eg, P-EBT^b^ and online SNAP purchasing) 3. Direct requests for help to pay bills and buy food | - “food” - “peopl” - “stamp” - “snap” - “need” - “can” - “get” - “homeless” - “unemploy” - “will” | 26,446 (20.48) | - “The right to eat doesn’t mean you can just go to the grocery store and get what you want and not pay champ. We have food stamps that people can apply for. We already have 1 party worried that providing food & shelter disincentivizes work we need 1 that understand what empathy is.” - “One of the most effective ways we can protect families from hunger is to #boostSNAP (formerly known as food stamps) & help more Americans enroll in the #SNAP program. Call your senator now to ask for an increase in SNAP benefits in the next #reliefbill #HungerActionMonth” - “I’m so fucking hungry. I have two cans of chicken and rice soup and some protein powder, and that’s it. I get food stamps on the first. Please can anyone spare a couple dollars for food? PayPal [username] Venmo CashApp [username]” |
| Grocery shopping and food workers: 44, 8, and 27 | 1. Discussions regarding the safety of shopping in person for at-risk populations (eg, special supermarket hours for older adults and shopping outdoors at farmers’ markets) 2. Discussions regarding the ethics of grocery delivery (eg, fair pay, tipping etiquette, and striking app delivery workers) 3. Discussions about barriers to shopping (eg, online use of EBT^c^ and increased cost of fresh fruits and vegetables) | - “instacart” - “food” - “groceri” - “worker” - “deliveri” - “get” - “order” - “store” - “can” - “time” | 20,515 (15.89) | - “Aldi launches home delivery service for vulnerable and self-isolating customers” - “Please stand by workers today as they strike for basic protections, sensible paid leave policies, and hazard pay to account for the inherent danger of working on the frontlines in a pandemic. Don’t buy from Amazon, Target, Instacart, or Whole Foods.” - “Uggggggg..i can’t get a food delivery from anyone till after Easter. Now Instacart going on strike. So many people going to grocery stores. I would go, but can’t chance it. I take care of my 90 year old mom. If I come down with it or even just become carrier noooooo goooood” |
| School foods: 15 and 16 | 1. Information sharing and announcements regarding P-EBT and grab-and-go school meals programs 2. Discussions regarding feeding food-insecure students during the pandemic | - “school” - “lunch” - “meal” - “food” - “student” - “Need” - “children” - “kid” - “day” - “can” | 16,023 (12.41) | - “Attention public-school families! NYC families are being sent P-EBT cards, regardless of income. Every single public-school student will receive $420 in P-EBT. If you don’t need it, these funds can be shared with food insecure families.” - Retweet: “On a typical school day, 21 million #K12 students from low-income backgrounds rely on free or reduced-price breakfast & lunch. With schools across the country closed indefinitely the U.S. is facing the risk of massive child hunger.” |
| Millions go hungry: 37, 18, and 39 | 1. Discussions about the state of food insecurity and unemployment because of the COVID-19 pandemic | - “food” - “insecur” - “pandem” - “need” - “peopl” - “new” - “covid” - “help” - “million” - “health” | 9103 (7.05) | - “Heartbreaking that millions of U.S. families experience hunger and food insecurity, and not just during the pandemic. Food banks, pantries & charities are struggling to meet the demand. Let’s do what we can to help & not look away.” |
| Food justice: 2 and 4 | 1. Discussions about the impact of climate change on health and food sources, grassroots efforts in addressing food security, food justice, and food waste | - “food” - “communiti” - “fresh” - “garden” - “produc” - “can” - “farm” - “distribut” - “qualiti” - “need” | 9004 (6.97) | - “There are many people working on the grass-roots level to help mitigate these issues. Many Black people are working in urban and rural agriculture to provide fresh quality produce and foods to communities in need.” |

^a^SNAP: Supplemental Nutrition Assistance Program.

^b^P-EBT: pandemic electronic benefit transfer.

^c^EBT: electronic benefit transfer.

**Multimedia Appendix 3**. Top 10 viral tweets, in chronological order of original post, presented with each tweet’s total number of likes and retweets at the time of data collection and a brief qualitative summary of the content posted in replies and quote tweets.

| Date | Tweet | Author | Number of followers | Number of likes^a^ | Number of retweets^a^ | Summary of replies and quote tweets |
| --- | --- | --- | --- | --- | --- | --- |
| March 9, 2020 | “What is wrong with us??? ‘Officials in NYC said they would close public schools only as last resort, in part because about 114,000 students in school system are homeless and may have nowhere else to get hot meals, medical care or even a place to wash their dirty laundry’” | Academician | 7744 | 46,253 | 15,195 | 1. Users expressing concern that schools are “petri dishes” and that keeping schools open will exacerbate the threat of COVID-19 2. Users discussing what would need to be present to ensure children would not miss out on the resources they receive at school (food, medical care, clean clothes, and counseling) 3. Feelings of shame for the state of our country |
| March 10, 2020 | “At some point, we have to talk about the fact that, in what is consistently touted as a thriving economy, the key concern w/closing schools b/c of COVID-19 is ‘children who rely on school food won’t get to eat.’ If the economy Is so great, why aren’t the babies eating?” | Nutritionist and personal trainer | 15,842 | 92,011 | 21,310 | 1. Comparisons of the “greatness” of the US economy for the 1% versus the working class 2. Information sharing regarding public libraries and schools that will continue distributing school meals during the lockdown 3. Mentions of the ripple effect that closing schools would have on working families and trouble accessing childcare |
| March 31, 2020 | “First Instacart, then Amazon, now Whole Foods: workers are withholding their labor& demanding dignity. When people work an hourly job, it’s suggested in many ways that you‘re unimportant or expendable. Except you aren’t. Everyone deserves safe work, paid leave, & a living wage.” | Politician | 6,843,060 | 46,133 | 10,600 | 1. Political arguments about socialism versus capitalism 2. Anger on behalf of people who need delivery service 3. Comparisons to if nurses were to strike during the pandemic |
| April 27, 2020 | “At a time when there are hungry New Yorkers, milk shouldn’t be dumped. We are partnering with NYS dairy producers to use the excess milk to make yogurt and cheese that will be distributed to food banks & those in need.” | Politician | 2,050,909 | 44,373 | 6946 | 1. Comments desiring the politician be elected President one day 2. Comparisons to other examples of excess food being distributed to people in need from other regions 3. Mentions of how a program such as this would help farmers |
| April 30, 2020 | “Amazon, Whole Foods, Instacart and I’m hearing Trader Joe’s and Target workers are striking TOMORROW, May 1st. Do NOT shop at any of these locations tomorrow, online or in person. It’s crossing a picket line. Never cross a picket line.” | Labor union activist | 956 | 24,794 | 11,925 | 1. Asking for clarification if the strike is New York City specific or nationwide 2. Users agreeing with the strike and urging others to not cross the picket line 3. Workers at these companies saying that they did not hear about the strike 4. Conversations about creating unions 5. Users saying they need the services and will use them anyway despite the strike |
| June 9, 2020 | “Hi. When republicans want to defund things like food stamps they just call it tax cuts. Happy to help.” | Political analyst | 251,447 | 298,839 | 68,784 | 1. Users promoting the “Defund the police” movement 2. Users hoping police funding can be reallocated to government assistance programs |
| June 11, 2020 | “Police departments get hundreds of millions of dollars a year, but children have ‘school lunch debt.’ Make it make sense.” | Fashion journalist | 54,271 | 171,569 | 67,605 | 1. Conversations about the cycle of poverty and comparisons to the prison system 2. Frustration with high police budgets compared to public school budgets 3. Encouragement toward free school lunch programs |
| October 7, 2020 | “Today in my IG story: How the Black Panthers’ People’s Free Food Program led to Free & Reduced School meal policies nationwide (and inspired #TeamAOC’s COVID food relief operation in 2020) 🥗” | Politician | 9,801,532 | 50,685 | 11,282 | 1. Thankyous for information sharing 2. Comments desiring the politician be elected President one day 3. Further information sharing about Black Panthers’ history with food programs, such as the Special Supplemental Nutrition Program for Women, Infants, and Children 4. Praise for free and reduced-price lunch in schools today 5. Mentions of the Black Panthers that are incarcerated |
| November 28, 2020 | “We did it! $200k raised in one livestream (on a whim!) for eviction defense, food pantries, and more. This is going to make such a difference for those who need it most right now. Thank you all 👾💞” | Politician | 10,793,716 | 150,923 | 9454 | 1. Users expressing gratitude that the politician is using their platform to help people in need 2. Users mentioning that charity is just a “band-aid” and does not address the root cause of food insecurity and other systematic issues |
| February 26, 2021 | “Preserving the filibuster is not worth letting millions of people in this country go hungry, sleep in their cars, or struggle to afford baby formula.” | Politician | 12,686,563 | 229,168 | 28,875 | 1. Users claiming that if power shifts in the Senate, the Democrats will want the filibuster back |

^a^These figures reflect these metrics when the data were collected from December 2021 through February 2022. It is possible that by the end of the time analyzed, March 31, 2021, these figures were lower and that more likes and retweets were added in the 7 months before the data were collected.
